# Malignant phyllodes tumor with metastases to lung, adrenal and brain: A rare case report

**DOI:** 10.1016/j.amsu.2018.10.030

**Published:** 2018-11-02

**Authors:** Suman Khanal, Yogendra P. Singh, Anuja Bhandari, Rashmi Sharma

**Affiliations:** a Breast and Thyroid Surgery Unit, Department of GI and General Surgery, Tribhuvan University Teaching Hospital, Nepal; b Department of Pathology, Tribhuvan University Teaching Hospital, Nepal; c Department of Dermatology, Tribhuvan University Teaching Hospital, Nepal

**Keywords:** Adrenal, Brain, Malignant, Metastasis, Phyllodes

## Abstract

**Background:**

Phyllodes tumors are spectrum of tumors ranging from benign to malignant. Malignant spectrum pose a management challenge for clinicians due to high risks of recurrence and metastasis. Malignant phyllodes tumor with brain, lung and adrenal metastases at the same time is rare.

**Case presentation:**

A 37 years-old unmarried female presented with an ulcerated huge lump in left breast with severe bleeding. Trucut biopsy showed necrosis with spindle cell proliferation with atypia for which she underwent modified radical mastectomy with final diagnosis of malignant phyllodes tumor. Three months after surgery, patient presented with headache, which on further evaluation showed masses in lung, right adrenal and brain.

**Conclusion:**

We presented a rare case of malignant phyllodes tumor with clinical, imaging and histological findings with metastases to multiple sites early in course and poor outcome of the patient despite margin negative resection.

## Highlights

• Metastatic malignant phyllodes tumor have poor outcome.
• Simultaneous metastasis to brain, lung and adrenal is rare.
• Margin negative resection is feasible even in huge malignant phyllodes tumor.

## 1 Background

Phyllodes tumors are uncommon fibroepithelial neoplasms of the breast that account for only 0.3–0.5% of primary breast tumors(1). They are classified by WHO into benign, borderline and malignant variants based on stromal cellularity, cellular atypia and pleomorphism, mitotic index, stromal overgrowth, tumor margin, and the presence or absence of heterologous differentiation [2]. The behavior of these tumors are unpredictable [1] and there is often no correlation between histological grading and clinical behavior due to intratumoral genetic heterogeneity [3]. In this report we present a rare case of bleeding giant malignant phyllodes tumor with metastases to brain, lung and adrenal in an unmarried female and review the literature. This work has been reported in line with SCARE criteria [4].

## 2 Case presentation

A 37 years-old unmarried premenopausal female presented in the emergency of our hospital with complaints of bleeding from ulcerative lesion in the left breast for a couple of hours. The patient had history of ulcer in the left breast for past 2–3 weeks which developed over the underlying huge breast lump. The lump was present for past 5 months but rapidly grew over the last 3 months to current size. There was no history of coagulopathy, trauma, chronic liver disease or history of similar illness in first degree relatives. The patient attained menarche at the age of 15 years. She is a non-smoker and denies history of contraceptive use or alcohol intake.

**Fig. 1:**
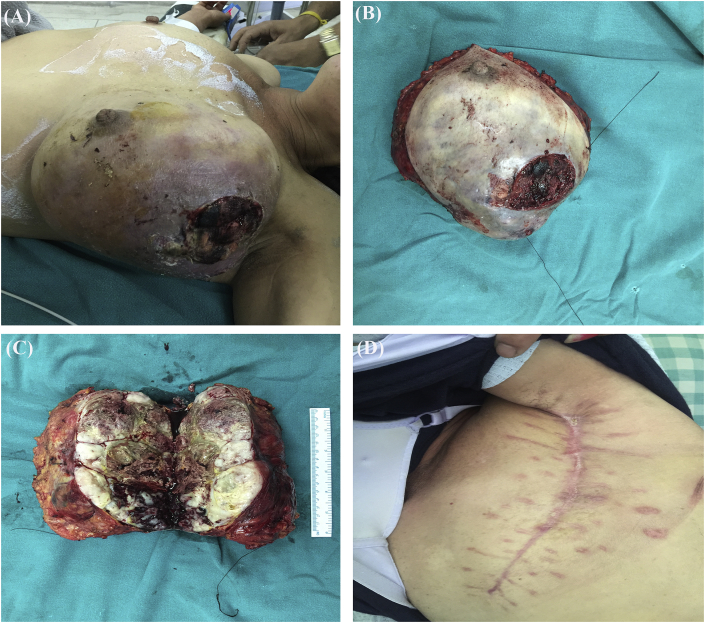
(a) Ulcerated lesion with huge lump in left breast (b) Resected mastectomy specimen (c) Cut section of the growth showing extensive necrosis (d) Three months postoperatively with no locoregional recurrence.

On examination in our emergency there was severe pallor with tachycardia. However the blood pressure was maintained. There was an oozing ulceration in upper outer quadrant of the left breast with underlying huge lump measuring 15 × 15 cm occupying the upper outer, lower outer and upper inner quadrants with deformed shape of the breast (Fig. 1a). The skin surrounding the ulcer was erythematous and edematous. Axilla did not show lymphadenopathy.

Before the development of breast lump, the patient had itchy lesion at the same site. The patient unfortunately attributed all these events to that itchy lesion and sought help late. There was no history of headache, shortness of breath, abdominal distension or bone pain.

**Fig. 2:**
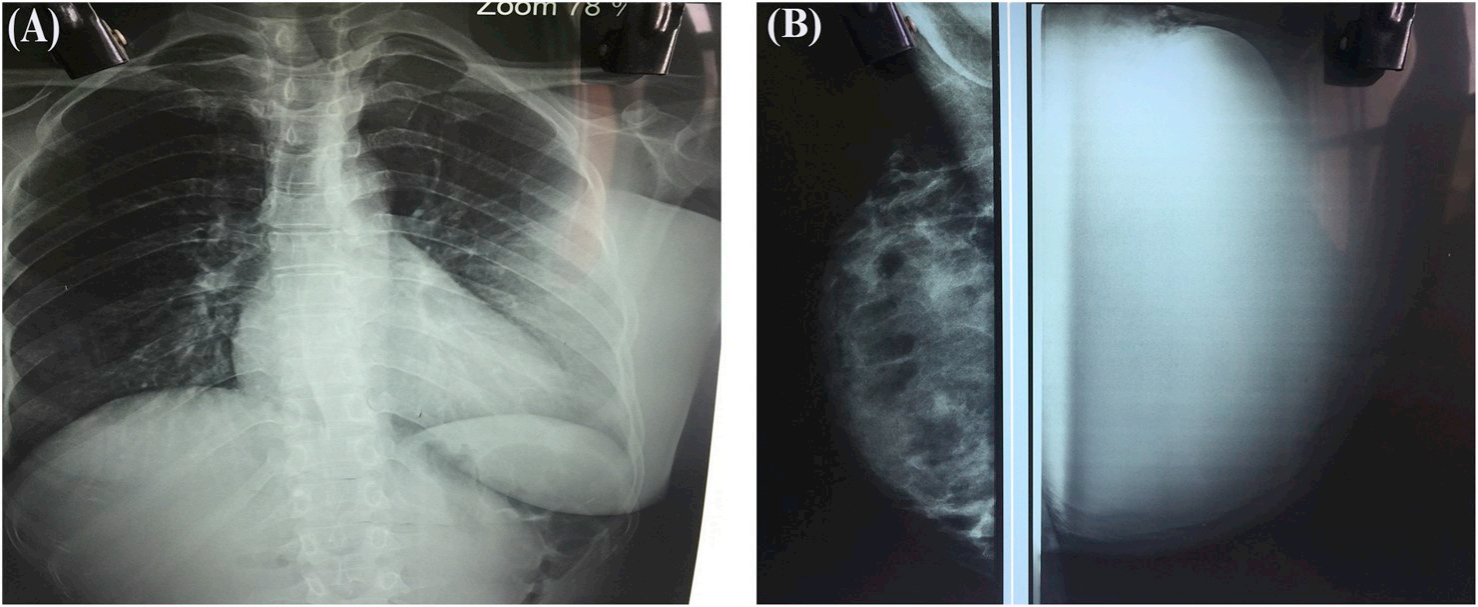
(a) Chest X-ray showing prominent breast shadow on left (b) Radiodense lobulated mass in mammogram without calcifications.

On investigating the patient, she was severely anemic with hemoglobin of 5 gm% with normal total and differential counts. Coagulation parameters and liver function tests were normal. Chest X-ray showed no evidence of metastasis other than dense breast shadow on left side due to huge breast lump(Fig. 2a).

Mammogram revealed homogeneous radiodense lobulated mass occupying whole of left breast with subcutaneous thickening on the background of dense breast (Fig. 2b). Ultrasound (USG) of abdomen and pelvis did not reveal any abnormal findings. Trucut biopsy from the breast mass showed necrotic areas with fibrocollagenous tissue with proliferation of oval to elongated spindle cells showing mild degree of atypia.

**Fig. 3:**
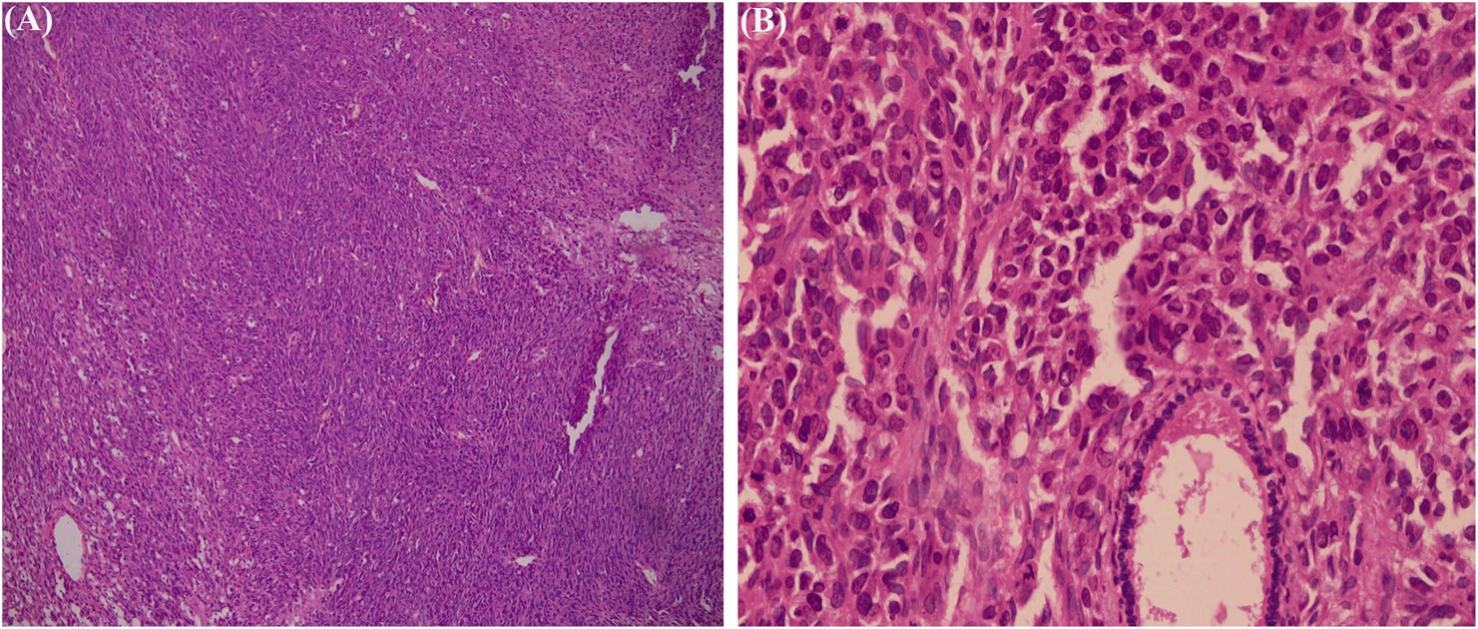
Hematoxyin and eosin (H&E) stain showing proliferation of stromal and ductal elements (a) 10X magnification (b) 40X magnification.

After discussion about the possibility of metaplastic versus malignant phyllodes tumor, the patient underwent modified radical mastectomy (MRM). Histopathological examination showed maximum tumor size of 14 cm with increased stromal cellularity, loss of stromal-epithelial balance and frequent mitoses more than 45 per 10 high power fields (Fig. 3) typical of malignant phyllodes. All the resected margins, nipple and areola were free of tumor. Lymphovascular and perineural invasions were not identified. Seventeen axillary nodes retrieved were free of tumor.

**Fig. 4:**
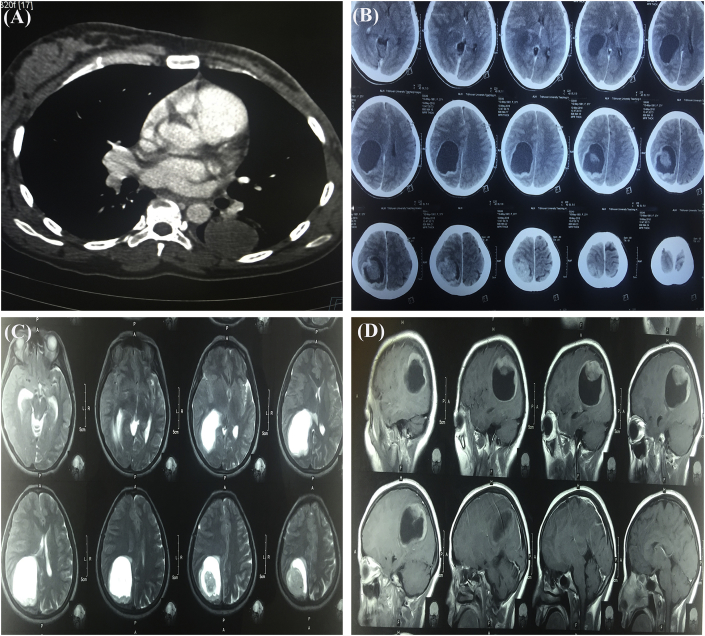
(a) Well defined round soft tissue density lesion of +40HU(Hounsfield unit) in posterobasal segment of left lower lobe of lung (b) CT scan of head showing well capsulated cystic lesion with heterogenously enhancing eccentric mural nodule with significant midline shift (c & d) T2 and T1 weighted MRI with Gadolinium contrast, respectively of head with enhancing wall, mural nodule and significant midline shift.

**Fig. 5:**
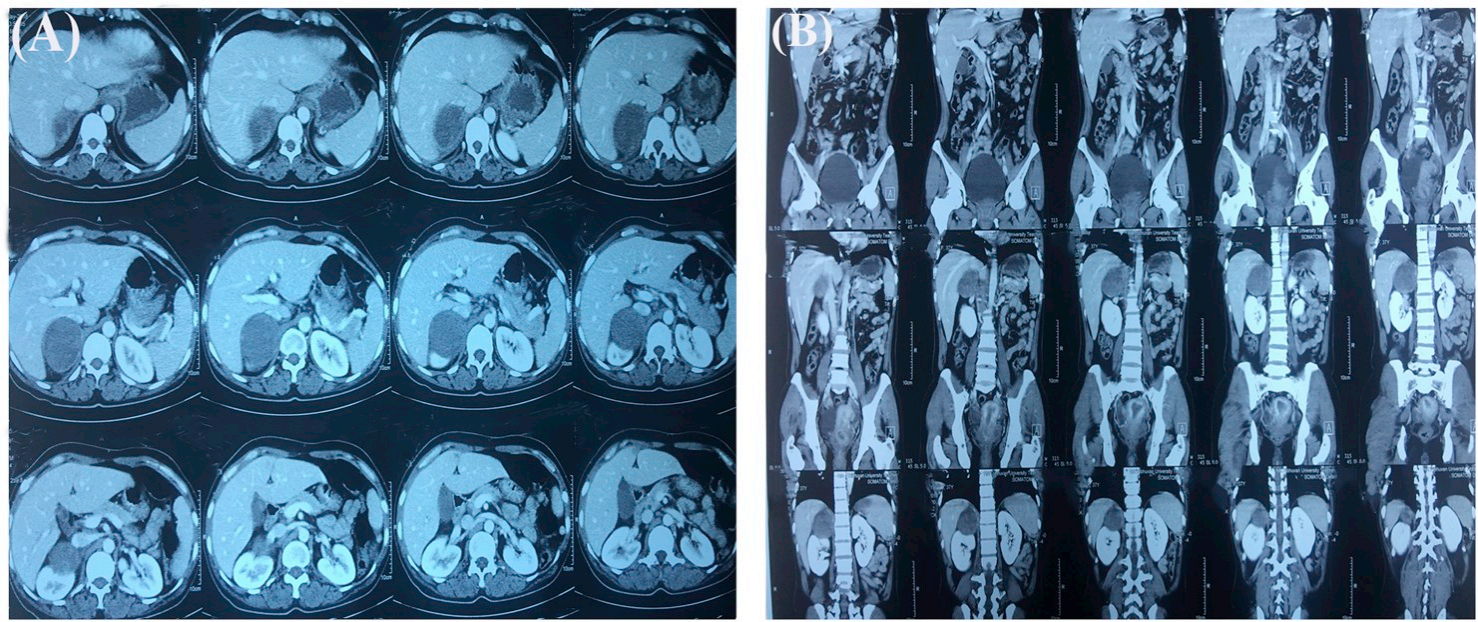
Cystic well defined lesion of +30HU in right suprarenal region with significant enhancement (a) Axial section (b) Coronal section.

Postoperatively patient gained weight and was doing well. Three months after surgery, the patient started developing progressively increasing headache, nausea and vomiting for which she got admitted in our center. There was no local recurrence. However on further investigations, she had a huge cystic lesion with mural nodule in her brain (Fig. 3b,c,d). Contrast enhanced computed tomography (CECT) of chest, abdomen and pelvis showed lesions in lower lobe of left lung (Fig. 4a) of size 3.9 × 3.6 cm of +40 Hounsfield unit (HU) in posterobasal segment and right adrenal gland (Fig. 5) of 5.8 × 5.1 cm of +30HU with significant enhancement in post-contrast images. Functional evaluation for the adrenal mass with urinary metanephrines and serum cortisol was negative. Patient refused biopsy from right adrenal gland and lung, but while preparing for burrhole biopsy from brain, patient succumbed to the disease. The patient underwent serial ultrasound of abdomen and pelvis during hospital stay which showed increase in size of 4 cm of adrenal mass in 15 days (tumor velocity).

**Figure:**
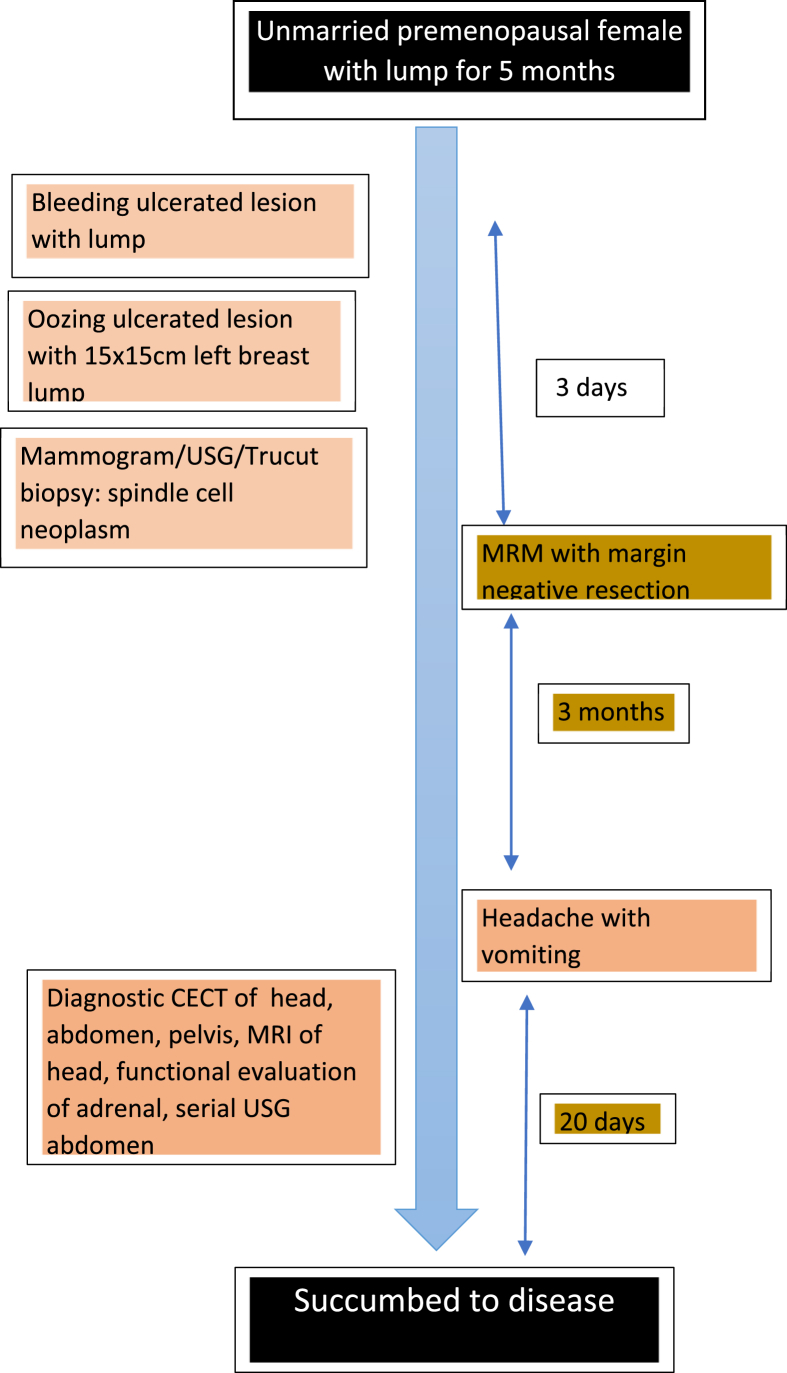
**Timeline**: Summary of timeline of events.

## 3 Discussion

Phyllodes tumors are notorious for local recurrence and progression. Only less than 10% of these tumors grow larger than 10 cm and the largest reported case happens to be of 50 × 50 cm by Islam et al. [5]. Approximately 85–90% of phyllodes tumors are benign and 10–15% are malignant [6] with metastasis in 10–26% cases of malignant phyllodes [7,8].

There are two cases of malignant phyllodes with simultaneous brain, lung and adrenal involvement [9]. Our case is unique in that the tumor was large >10 cm which itself is rare with few reports [10]. Though lungs and bone are common sites of metastasis in malignant phyllodes tumors [11], our patient had metastasis to adrenal and brain which is rare. The duration of metastasis after surgery is 7 months to 6 years in literature [9], however our case developed it early in 3 months. Large tumor size and malignant phyllodes tumor are shown to be predictive of metastasis [12]. It thus explains the metastasis in our case.

Patient attributed the illness to itching and sought treatment late when she already had ulceration and bleeding from the lesion. Increasing awareness is required especially in developing countries where health seeking behaviors are still poor and where people correlate ominous diseases with some trivial event that does not even have temporal relationship with the current illness.

Closure of wound after excision of giant phyllodes tumor is a challenge. However total mastectomy with primary closure is still feasible in such cases while achieving negative margins as we did in our case. We do not image the head and spine routinely unless the patient is symptomatic in these areas. Our practice to screen for metastasis is to image abdomen and pelvis with ultrasound, and chest with X-ray first, proceeding to CT if any abnormalities are detected in these scans.

One may argue that the lesions in chest or abdomen could be present during initial presentation although the chest X-ray and USG abdomen and pelvis were negative. But given the rapid increase in size of adrenal (tumor velocity), we strongly believe that there could not have been such lesions during initial presentation.

We believe that atleast chest and abdomen should be screened with contrast enhanced CT especially in cases with malignant phyllodes tumor so as not to miss subtle lesions even if the patient is asymptomatic at these sites.

Postsurgical management in margin negative phyllodes tumor is still controversial. A study with 30 malignant phyllodes cases do not recommend radiotherapy in margin negative resection [7]. Although use of radiotherapy has increased recently due to high risk of recurrence, there is no level 1 data supporting such practice [13]. Use of chemotherapy is similarly controversial after primary margin negative resection though they are seeing increased use in palliation in metastatic cases. Our patient did not receive radiotherapy or chemotherapy due to this controversy and indeed there was no locoregional recurrence.

## 4 Conclusion

Malignant spectrum of phyllodes is notorious for metastases with dismal outcome. Although the lungs and bones are the most common sites, there can be simultaneous metastases to brain, lung and adrenal as in our case. Contrast enhanced CT of chest and abdomen should be routinely done in malignant phyllodes tumors to look for metastasis.

## Ethics approval and consent to participate

Not applicable as it is a case report.

## Funding

No funding was received from any source for this work.

## Authors’ contributions

SK wrote the article. YPS and SK were the operating surgeons, AB and RS helped in literature review, acquisition of histological, radiological images and revising the manuscript. All authors read and approved the final manuscript.

## Conflicts of interest

None.

## Registration of research studies

Not applicable.

## Guarantor

Suman Khanal, Yogendra P Singh.

## Consent for publication

Written informed consent was obtained from the patient for publication of this case report and accompanying images. A copy of the written consent is available for review by the Editor-in-Chief of this journal on request.

## Availability of data and material

Not applicable as it is a case report.

## Abbreviations

USG: Ultrasound
MRM: modified radical mastectomy
CECT: contrast enhanced computed tomography
MRI: magnetic resonance imaging
HU: Hounsfield unit

## Appendix A Supplementary data

SCARE_checklist SCARE_checklist
