# Supplementary material for: Malignant phyllodes tumor with metastases to lung, adrenal and brain: A rare case report
Source: Ann Med Surg (Lond). 2018 Nov 2;36:113–7. doi: 10.1016/j.amsu.2018.10.030 (PMC6230968; doi:10.1016/j.amsu.2018.10.030)
Supplement: SCARE_checklist [file mmc1.docx]

CARE checklist

**Title:** separate file

**Keywords:** Page 1

**Abstract:**

**3a** Page 1, paragraph 1

**3b** Page 1, paragraph 2

**3c** Page 1, paragraph 2

**3d** Page 1, paragraph 3

**Introduction:** Background, Page 1

**Patient information:**

**5a** Case Report, Page 2, paragraph 1

**5b** Case Report, Page 2, paragraph 1

**5c** Case Report, Page 2, paragraph 1

**5d** N/A

**Clinical findings:** Case Report, Page 2, paragraph 2-3

**Timeline:** timeline file separate

**Diagnostic assessment:**

**8a** Case Report, Page 2, paragraph 4-5

**8b** Case Report, last paragraph

**8c** Case Report, Page 2, paragraph 6

**8d** Case Report, Page 2, paragraph 7

**Therapeutic Intervention:**

**9a** Case Report, Page 2, paragraph 6

**9b** N/A

**9c** N/A

**9d** N/A

**9e** N/A

**9f** N/A

**Follow up and outcomes:**

**10a** N/A

**10b** Case Report, Page 2, last paragraph

**10c** N/A

**10d** N/A

**Discussion:**

**11a** Page 3, paragraph 4-6

**11b** Page 3, paragraph 1-2

**11c** Page 3, paragraph 1-2

**11d** Page 3, paragraph 6-7

**Patient perspective:** N/A

**Informed consent:** Page 4

**Additional information:** Declarations, page 4
